# Integration of Artificial Intelligence in Medicines

**DOI:** 10.31662/jmaj.2024-0080

**Published:** 2024-06-28

**Authors:** Masaki Mori

**Affiliations:** 1 Tokai University School of Medicine, Isehara, Japan

**Keywords:** Artificial Intelligence, Medicine, Machine Learning, Deep Learning, Neural Network

Artificial intelligence (AI) has transformed numerous sectors, including medicine ^(1), (2)^, with recent enhancements in machine learning with neural networks. Given the ability to analyze large, complex datasets, AI can enable the handling of information beyond humans’ understanding. This potential has been supported by recent studies showing that AI is capable of detecting diseases and predicting prognosis beyond the ability of fully trained experts ^(3)^. However, when adopting this useful technology, we must be aware of its merits and limitations.

One of the most significant benefits of AI in medicine is its ability to improve diagnostic accuracy, which sometimes exceeds fully trained experts. For example, AI algorithms trained to detect atrial septal defects from 12-lead electrocardiograms displayed performances far beyond the readings of cardiologists ^(4)^. Along with other AIs technologies that show equal to higher performance compared to experts, medical imaging is one of the fields to embrace the early integration of AI.

The other strength of AI is its ability to extract features without the need for a human to teach them. However, these characteristics can introduce some confusion. While the training process may sound simple, there are various factors that need to be considered when training a useful model. One such factor is the black-box nature of AI models: not being able to explain why they predict what they predict. This is a well-known weakness of AI that occasionally results in the development of useless models with unwanted features. A prominent example is the use of scanner types in an AI model trained with X-ray images. A model trained to detect pneumonia from chest X-ray images showed apparently high discrimination, however, eventually, it was found to have a fundamental flaw: the model used the settings where the X-ray was obtained to calculate the risk of pneumonia. As those who had an X-ray taken at inpatient settings with a portable scanner had a significantly higher prevalence of pneumonia, the model presumably used the difference in setting as a feature to detect pneumonia. This example suggests that we must be cautious about how the model can “cheat.” In the author’s opinion, there are at least two crucial aspects for preventing the AI model from “cheating”: (1) deep understanding of the data and (2) rigorous validations using external datasets.

AI’s ability to automatically extract features from data is sometimes misunderstood as a belief that AI models can be built without understanding the data. This is an enormous misunderstanding. This characteristic means that the AI model may use any features present in the data to calculate the prediction. As mentioned above, this easily leads to useless models. To avoid the model from using “false” features, the researcher needs to carefully engineer the dataset such that the “false” features will not be informative. This is usually done by removing image contents outside of a boundary or by balancing between cases and controls.

Even after carefully engineering the training dataset, there can still be unrecognized features that could be used by the AI to “cheat.” Moreover, the AI model may perform well within some specific populations within the dataset and poorly in others. To account for these residual unwanted features and heterogeneity across subgroups, rigorous validations using external datasets becomes important. In most cases, the “false” features are artifacts introduced during data collection. It is conceivable that different institutions with different technology that use different equipment, vendors, or models for obtaining the data do not share these artifacts. Thus, if the AI model performs similarly on an external validation with a diverse dataset, it is unlikely that the model is using “false” features. By combining external validation with rigorous subgroup analysis, we can identify the models using “false” features and understand the boundary of applicability of AI modes ^(5)^.

In conclusion, the future of AI in medicine is promising but requires careful consideration of the aforementioned challenges. Large-scale collaborations across multiple institutions, along with those across fields (e.g., AI developers and healthcare professionals) to enhance rigorous validations and deep understanding of the data, are crucial for creating AI models applicable to medicine. Ongoing research and development is essential to improve the capabilities of AI models in medicine. This should be coupled with efforts to improve the quality and diversity of the datasets used to train AI models, thereby reducing the risk of bias and improving outcomes across diverse patient populations. The implementation of AI models in medicine is still at the beginning stage. Continuous research is still warranted in this field. JMA Journal welcomes submissions of AI articles, while publishing commissioned papers. In so doing, please read the *Use of Artificial Intelligence (AI)-Assisted Tools/Technologies* in the Instructions for Authors.

## Article Information

### Conflicts of Interest

None

### Disclaimer

Masaki Mori is one of the Associater Editors of JMA Journal and on the journal’s Editorial Staff. He was not involved in the editorial evaluation or decision to accept this article for publication at all.
